# [Retracted] Galectin‑3 blockade suppresses the growth of cetuximab‑resistant human oral squamous cell carcinoma

**DOI:** 10.3892/mmr.2024.13275

**Published:** 2024-07-01

**Authors:** Peng Yin, Shuanlong Cui, Xiangling Liao, Xiaoguang Yao

Mol Med Rep 24: 685, 2021; DOI: 10.3892/mmr.2021.12325

Following the publication of this paper, it was drawn to the Editor's attention by a concerned reader that the colony formation assay data shown in Fig. 4C on p. 6 were strikingly similar to data appearing in different form in other articles written by different authors at different research institutes, which had already been published. Owing to the fact that the contentious data in the above article had already been published prior to its submission to *Molecular Medicine Reports*, the Editor has decided that this paper should be retracted from the Journal. After having been in contact with the authors, they accepted the decision to retract the paper. The Editor apologizes to the readership for any inconvenience caused.

